# Volatile Constituent Analysis of Wintergreen Essential Oil and Comparison with Synthetic Methyl Salicylate for Authentication

**DOI:** 10.3390/plants11081090

**Published:** 2022-04-17

**Authors:** Pawan Kumar Ojha, Darbin Kumar Poudel, Sabita Dangol, Anil Rokaya, Sujan Timsina, Prabodh Satyal, William N. Setzer

**Affiliations:** 1 Analytica Research Center, Kritipur, Kathmandu 44660, Nepal; pojha@aromaticplant.org (P.K.O.); darkwine51@gmail.com (D.K.P.); sdangol@aromaticplant.org (S.D.); arokaya@aromaticplant.org (A.R.); stimsina@aromaticplant.org (S.T.); 2 Aromatic Plant Research Center, 230 N 1200 E Suite 100, Lehi, UT 84043, USA; 3 Department of Chemistry, University of Alabama in Huntsville, Huntsville, AL 35899, USA

**Keywords:** adulteration, dimethyl 2-hydroxyterephthalate, enantiomeric ratio, synthetic marker, *Gaultheria*

## Abstract

A comparative analysis of *Gaultheria fragrantissima* (Ericaceae) essential oils based on geographical location, distillation time, and varying distillation conditions was carried out, and their compositions were evaluated by gas chromatography–mass spectrometry (GC–MS), chiral GC–MS, and gas chromatography–flame ionization detection (GC–FID). In addition, each of seven commercial wintergreen essential oil samples from Nepal and China were analyzed. The highest extraction yield was 1.48% and the maximum number of compounds identified in natural wintergreen oil was twenty-two. Based on distillation time, the maximum numbers of identified compounds are present in 120 min. Linalool, phenol, vetispirane, and ethyl salicylate were present in commercial wintergreen oils both from Nepal and China. The presence of compounds such as elsholtzia ketone and β-dehydroelsholtzia ketone in the China samples represented a significant difference in wintergreen oil between the two geographical sources. Dimethyl 2-hydroxyterephthalate is a well-known synthetic marker for wintergreen oil when synthesis is carried out using salicylic acid, but the synthetic marker was absent while using acetylsalicylic acid as a precursor during synthesis. Adulteration analysis of wintergreen oil showed an increase in the concentration of dimethyl 2-hydroxyterephthalate, whereas the concentrations of minor components decreased and methyl salicylate remained unchanged. To the best of our knowledge, this is the first report of the enantioselective analysis of wintergreen essential oil. Furthermore, three samples showed notable antibacterial activity against *Staphylococcus epidermidis*, with an MIC value of 156.3 μg/mL. Similarly, one sample showed effectiveness against *Aspergillus niger* (MIC = 78.1 μg/mL).

## 1. Introduction

*Gaultheria fragrantissima* Wall, commonly known as “dhasingre” in Nepal, is an evergreen perennial shrub growing at an altitude of 1800–2500 m that belongs to the family Ericaceae, which comprises around 200 different species; among these, *G. fragrantissima* of Indo-Nepal origin and *Gaultheria procumbens* L. from North America are the most popular species for the production of wintergreen essential oils. The essential oils are generally produced by steam or hydrodistillation, and during the distillation, the glycoside gaultherin present in the wintergreen plant is converted to methyl salicylate. It is the major compound above 98% present in wintergreen oil and is used in food industries, pharmaceuticals, cosmetics, and aromatherapy. With the increasing demand in different industries and cost of natural wintergreen oil, there is always a chance of adulteration by synthetic methyl salicylate [1,2,3]. Since it can be easily synthesized from salicylic acid or its acetyl derivative aspirin by following the Fisher esterification reaction, the acid-catalyzed acyl substitution of the carboxylic acid with the nucleophilic alcohol can be performed to give an ester [4]. Different works have been carried out for the detection of adulteration in wintergreen oil by using different techniques such as biomarker-based analysis, ^14^C radiocarbon activity [5], isotope ratio mass spectrometry (IRMS) [6], and the most recent technique, site-specific natural isotope fractionation by nuclear magnetic resonance (SNIF-NMR) [7].

In previous studies, researchers have abandoned chiral GC–MS for the authentication of wintergreen oil, since the major compound present is an achiral molecule, but they neglect the other minor compounds present in the oil. However, in this work, we have considered these minor compounds and carried out the chiral GC–MS of wintergreen oil. The enantiomeric ratio of different chiral compounds determined by chiral GC–MS is used for the authentication of purity and origin of essential oils [8].

This study aims to develop a reliable technique for the detection of adulteration or authentication of wintergreen oil from Nepal. Samples were collected from different geographical locations of Nepal and a comparison was made with natural versus manufactured and commercial methyl salicylate. GC–MS/GC–FID and chiral GC–MS were carried out for the development of a novel methodology for authentication of wintergreen essential oil as well as consumer reliability and safety.

## 2. Results and Discussion

### 2.1. Yields of Natural and Synthetic Wintergreen Oil

*Gaultheria fragrantissima* samples collected from four different geographical locations of Nepal were hydrodistilled with and without soaking before distillation along with the addition of salt, and the percentage yields are tabulated in Table 1. The highest extraction yield was observed in dry leaves (W6), at 1.48%, whereas for fresh leaves (W5), the yield increased drastically by soaking in 5% sodium chloride solution for 18 h (W8). The increased yield in the soaked sample and salt-added sample can be attributed to the longer time period for hydrolysis, and the salt helps in the cleavage of the gaultherin molecule which finally converts to methyl salicylate [1]. Methyl salicylate synthesized from salicylic acid has the highest yield of 66.67% compared to methyl salicylate synthesized from aspirin tablets, which can be attributed to the titanium dioxide coating present as an impurity in aspirin tablets.

### 2.2. GC–MS Analysis of Natural Wintergreen Oil Based on Geographical Location

The results of GC–MS analysis of wintergreen essential oil from the different geographical locations Solukhumbu (W1), Syangja (W2), Champadevi (W3), and Sundarijal (W4) of Nepal are presented in Table 2. The major compound was methyl salicylate, an ester of salicylic acid which was above 99.50%, with a total of 4, 18, 22, and 6 compounds identified from W1, W2, W3, and W4, respectively. Here, essential oil W4 shows the highest number of compounds present, though they are in trace amounts, followed by W2 and W3, whereas essential oil W1 shows the lowest number of compounds present, since the chemical composition of essential oil can vary by geographical location. Along with methyl salicylate as the major compound, eugenol, a phenolic compound, and the monoterpene alcohol linalool are two other compounds that were present in all samples from different geographical locations.

### 2.3. GC–MS Analysis of Natural Wintergreen Oil Based on Distillation Time

The results of GC–MS analysis of wintergreen essential oil based on distillation time are presented in Table 3. The total compounds identified are 17, 20, 21, and 15 at a distillation time of 30, 60, 120, and 180 min, respectively. The maximum numbers of identified compounds are present in 120 min of distillation time, which indicates the optimum time to obtain the maximum number of compounds in wintergreen oil. In addition, with increasing time, there is slightly decreased methyl salicylate percentage which ultimately increases minor compounds such as linalool and manoyl oxide. Although there is a slight change in concentrations of minor compounds, no significant changes were observed.

### 2.4. GC–MS Analysis of Natural Wintergreen Oil Based on Different Distillation Conditions

The results of GC–MS and GC–FID analysis of essential oils derived from fresh (W5MS and W5FID) and dry (W6MS and W6FID) leaves, along with 18 h soaked (W7MS and W7FID) and 18 h soaked in 5% sodium chloride solution (W8MS and W8FID) are presented, respectively, in Table 4. A total of 22, 26, 13, and 17 compounds are identified for W5, W6, W7, and W8, respectively. Here, the major compound methyl salicylate was above 99% in each sample, and some changes were observed for some of the minor compounds. (3*Z*)-Hexenol was high in W5 compared to W6, but eugenol was high in W6 compared to W5, while ethyl benzoate was absent in W6. Limonene, 1-hexanol, linalool, nonanal, and ethyl salicylate were present in all four samples, while ethyl salicylate was present in the highest amount in W7 compared to the others. In addition, no significant difference in GC–MS and GC–FID results was observed.

### 2.5. GC–MS Analysis of Commercial Wintergreen Oil from Nepal and China

Commercial wintergreen essential oils from Nepal and China (seven each) were obtained from the Aromatic Plant Research Center (APRC) to compare their respective chemical compositions and variations, which are shown below in Table 5. The GC–MS report from APRC showed methyl salicylate to be the major compound, up to 99.86% in Nepalese oil, and up to 99.91% in Chinese wintergreen oil, which does not show drastic variation. Minor compounds such as linalool, phenol, vetispirane, and ethyl salicylate were present in both samples with slight variations, but compounds such as elsholtzia ketone and β-dehydroelsholtzia ketone were detected only in the Chinese commercial wintergreen oil, giving an important difference between the wintergreen oil of the two sources. 

### 2.6. GCMS Analysis of Synthetic Methyl Salicylate

The results of GC–MS analysis of commercial methyl salicylate (W11) and synthesized methyl salicylate using different starting materials are presented in Table 6. Methyl salicylate synthesized by using salicylic acid (W10) as a precursor shows almost the same result as commercial methyl salicylate with the presence of synthetic markers such as dimethyl 2-hydroxyterephthalate and methyl paraben, which are side products formed during the synthesis, while methyl salicylate synthesized using aspirin as the precursor (W9) looks somewhat different in chemical composition with the presence of ethyl salicylate, which was rarely present in synthetic samples but commonly found in natural wintergreen oil. In addition, there is an absence of the synthetic marker dimethyl 2-hydroxyterephthalate, which should be present in W9 but was not detected, while W10 and W11 show the presence of this compound. This creates a loophole for adulteration in natural wintergreen oil using synthetic methyl salicylate, and adulteration detection using GC–MS becomes difficult. Thus, another technique such as the ^14^C test can be performed to detect adulteration [5].

### 2.7. Comparison of GC–MS Analysis of Natural and Adulterated Wintergreen Oil

The results of GC–MS analysis of natural wintergreen oil with the addition of different percentages of commercial methyl salicylate samples are presented in Table 7. By increasing the concentration of synthetic methyl salicylate in wintergreen oil, most of the naturally occurring minor compounds such as α-pinene, 1-hexanol, 1,8-cineole, limonene, linalool, vitispirane, and β-dehydroelsholtzia ketone decreased with an increase of dimethyl 2-hydroxyterephthalate, indicating the adulteration in the oil, whereas concentration of methyl salicylate remained nearly constant. Thus, by using GC–MS analysis, addition of synthetic methyl salicylate with salicylic acid as a precursor can be detected.

### 2.8. Enantiomeric Distribution Analysis

Wintergreen oil is easily adulterated by adding synthetic methyl salicylate [9], which is detected by the presence of synthetic markers [10], but apart from synthetic markers, enantiomeric ratio determination is a powerful tool in authenticity establishment, especially when the enantiomeric forms are unchanged by the extraction processes and the acid index [8]. To the best of our knowledge, this is the first analysis of the enantiomeric distribution of chiral compounds present in wintergreen essential oils. There are altogether six chiral compounds detected in wintergreen essential oils, namely, linalool, α-terpineol, β-caryophyllene, α-pinene, limonene, and camphor, which are listed in Table 8. This shows that wintergreen has nearly racemic linalool and predominantly (−)-α-terpineol. Other components, such as (−)-β-caryophyllene (100%), were only detected in the sample from W3. (+)-Camphor was 100% in the samples of W1 and W4. (+)-α-Pinene and (+)-limonene were pure enantiomers detected from the samples of W1 and W2. Table 8 summarizes the enantiomeric compositions for different geographical locations in Nepal.

Likewise, Table 9 shows enantiomeric distributions of chiral terpenes of wintergreen essential oil based on distillation time, which reveals a total of four chiral molecules among which linalool and (−)-α-terpineol were detected and (−)-β-caryophyllene and (+)-camphor were detected as pure enantiomers. These variations in enantiomeric distribution may be because all the chiral components detected are in minor amounts, so it should not be considered as evidence of adulteration unless further proper research and investigation are carried out. However, these experiments show that geographical locations and distillation time do not affect the enantiomeric distribution of the chiral compounds present in wintergreen oil.

Similarly, Table 10 shows enantiomeric distributions of chiral compounds of W5, W6, W7, and W8, which show six chiral terpenes among which linalool and (−)-α-terpineol were similar to the previous samples, but terpenes such as (+)-camphene, (+)-limonene, (+)-β-phellandrene, and (+)-germacrene D were not detected in the above samples. Pure (+)-limonene was present in all samples, whereas 100% (+)-camphene and (+)-β-phellandrene were detected in W5, and 100% (+)-germacrene D was detected in the W7 sample. 

Correspondingly, Table 11 explains the enantiomeric distribution of commercial wintergreen oils from Nepal and China which detected six chiral terpenoids among which linalool, (−)-α-terpineol, and (+)-α-pinene were detected in both samples, but 100% (+)-camphor, (−)-β-caryophyllene, and (−)-β-Pinene were detected only in Nepalese wintergreen oil, which highlights the geographical variation in oils of both sources and that GC–MS alone was unable to elaborate.

### 2.9. Antimicrobial Activity

This study showed that the assayed wintergreen essential oil has different degrees of microbial inhibitory activity against the panel of bacteria, consistent with the observation that essential oils generally show selective activity against microorganisms [11]. The minimum inhibitory concentrations (MICs) of the wintergreen essential oil samples from Solukhumbu (W1), Champadevi (W3), and Sundarijal (W4) against a panel of bacteria and fungi are presented in Table 12 and Table 13, respectively. All tested essential oil samples showed better activity against *Staphylococcus epidermidis*, with an MIC value of 156.3 μg/mL. The essential oils of wintergreen demonstrated weaker antibacterial activities than those of the positive control gentamicin (MIC < 19.5 μg/mL), similar to a previous study of different essential oils along with wintergreen oil that showed low antibacterial activity [12].

The sample W1 showed effectiveness against *Aspergillus niger* (MIC = 78.1 μg/mL) compared to W3 and W4, while W1 and W3 showed effectiveness against *Microsporum canis,* and W4 against *Trichophyton mentagrophytes,* and W3 against *Trichophyton rubrum* with (MIC = 156.3 μg/mL). Wintergreen essential oils demonstrated weaker antifungal activities than those of the positive control, amphotericin B (MIC < 19.5 μg/mL) [13].

## 3. Materials and Methods

### 3.1. Sample Collection

**Figure 1:**
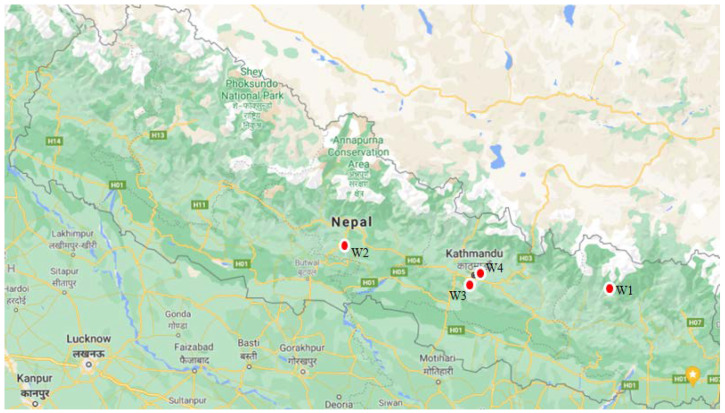
Collection sites of *Gaultheria fragrantissima* leaves.

*Gaultheria fragrantissima* Wall leaves were collected in 2021 from Solukhumbu (W1) (27°28′50.9″ N, 86°53′31.0″ E) at an elevation of 1875 m, Syangja (W2) (28°07′47.4″ N, 83°46′04.7″ E) at an elevation of 1700 m, Champadevi (W3), southwestern part of Kathmandu valley (27°38′19.3″ N, 85°15′44.7″ E) at an elevation of 2280 m, and Sundarijal (W4), northeastern part of Kathmandu valley (27°47′24.9″ N, 85°25′41.6″ E) at an elevation of 1620 m (Figure 1). In addition, a comparison was made between fresh leaves (W5) and dry leaves (W5) and by varying hydrodistillation conditions, 18 h soaked (W7) versus 18 h soaked in 5% NaCl solution (W8) with the sample collected from Champadevi. For 3 h, leaves of *G. fragrantissima* and water in 1:2 ratios (*w*/*v*) were hydrodistilled using a Clevenger apparatus. The essential oil was dried with anhydrous sodium sulfate and was stored in bottles at 5 °C until further analysis.

### 3.2. Synthesis of Methyl Salicylate

Synthetic methyl salicylate was prepared using two different precursors: salicylic acid (W10) (Sigma-Aldrich, St. Louis, MO, USA) [14] and aspirin tablets (W9) (USV Private Ltd., Mumbai, India) [4], where these precursor compounds were dissolved in methanol in 1:2 *w*/*v* ratios and were refluxed in a round bottom flask for around 90 min using sulfuric acid. After refluxing, the organic layer was separated from the aqueous layer and then it was cooled and excess sulfuric acid was neutralized using saturated sodium bicarbonate solution. The organic layer containing methyl salicylate was extracted using dichloromethane as a solvent, pure methyl salicylate was obtained by fractional distillation at around 222 °C, and yields were recorded. One commercial synthetic methyl salicylate (W11) sample from Thermo Fisher Scientific (Waltham, MA, USA) was also analyzed.

### 3.3. Volatile Composition Analysis by Gas Chromatography–Mass Spectrometry (GC–MS)

Natural wintergreen oil samples based on geographical location as well as distillation time, in addition to synthetic methyl salicylate, were analyzed using a Shimadzu GC–MS-QP2010 Ultra (Shimadzu Scientific Instruments, Columbia, MD, USA) with electron impact (EI) mode with 70 eV, along with ZB-5 ms capillary GC column, using 40–400 *m*/*z* range scans with a scan rate of 3.0 scan/sec. The column temperature was set at 50 °C for 2 min and then increased at 2 °C/min to the temperature of 260 °C. The carrier gas was helium with a constant flow rate of 1.37 mL/min. The injector temperature was kept at 260 °C. For each essential oil sample, 1:10 *v*/*v* solution in dichloromethane (DCM) was prepared, and 0.3 μL was injected using a split ratio of 1:30. Identification of the individual components of the essential oils was determined by comparison of the retention indices and comparison of the mass spectral fragmentation patterns (over 80% similarity match) with those found in the MS databases using the Lab Solutions GCMS post-run analysis software version 4.45 (Shimadzu Scientific Instruments, Columbia, MD, USA) [15,16,17].

### 3.4. Volatile Composition Analysis by Gas Chromatography–Flame Ionization Detection (GC–FID)

Analysis of natural wintergreen essential oil was carried out using a Shimadzu GC 2010 equipped with a flame ionization detector (Shimadzu Scientific Instruments, Columbia, MD, USA), as previously described [18], with ZB-5 capillary column (Phenomenex, Torrance, CA, USA).

### 3.5. Enantiomeric Analysis by Chiral Gas Chromatography–Mass Spectrometry (CGC–MS)

A Shimadzu GCMS-QP2010S with EI mode (70 eV) and B-Dex 325 chiral capillary GC column was used to perform enantiomeric analysis of wintergreen oil. Scans were in the 40–400 *m*/*z* range at a scan rate of 3.0 scan/sec. The column temperature was set at 50 °C, at first increased by 1.5 °C/min to 120 °C and then 2 °C/min to 200 °C. The final temperature of the column was 200 °C and was kept constant. The carrier gas was helium with a constant flow rate of 1.8 mL/min. For each essential oil sample, 3% *w*/*v* solution in DCM was prepared, and 0.1 μL was injected using a split ratio of 1:45 [15,16,18]. The enantiomer percentages were determined from the peak area. Comparison of retention times and mass spectral fragmentation patterns with authentic samples obtained from Sigma-Aldrich (Milwaukee, WI, USA) was used to identify the enantiomers.

### 3.6. Bacterial Strains Tested

Seven microorganisms were used to evaluate the antibacterial activities of some selected wintergreen essential oils: five Gram-positive bacteria, *Bacillus cereus* (ATCC-14579), *Staphylococcus epidermidis* (ATCC-12228), *Propionibacterium acnes* (ATCC-11827), *Staphylococcus aureus* (ATCC-29213), and *Streptococcus pyogenes* (ATCC-19615), and two Gram-negative bacteria, *Serratia marcescens* (ATCC-14756) and *Pseudomonas aeruginosa* (ATCC-27853), using the microbroth dilution technique. Tryptic soy agar medium was used to culture all tested bacterial strains. A 5000 μg/mL solution of wintergreen essential oil was prepared in dimethyl sulfoxide (DMSO) and twofold dilution in 100 μL of cation-adjusted Mueller Hinton broth (CAMHB) (Sigma-Aldrich, St. Louis, MO, USA) was added to the top well of a 96-well microdilution plate. The prepared stock solution of essential oils was then serially two-fold diluted in fresh CAMHB to obtain final concentrations of 2500, 1250, 625, 312.5, 156.3, 78.1, 39.1, and 19.5 μg/mL. The freshly harvested bacteria with approximately 1.5 × 10^8^ CFU/mL final concentration were added to each well of 96-well microdilution plates and were incubated at 37 °C for 24 h. Gentamicin (Sigma-Aldrich, St. Louis, MO, USA) and DMSO were used as positive and negative controls, respectively [15,19].

### 3.7. Fungal Strains Tested

Seven fungal strains were used: *Aspergillus niger* (ATCC-16888), Candida albicans (ATCC-18804), Microsporum canis (ATCC-11621), Trichophyton mentagrophytes (ATCC-18748), Aspergillus fumigatus (ATCC-96918), Microsporum gypseum (ATCC-24102), and Trichophyton rubrum (ATCC-28188). All tested fungi were cultured in a yeast–nitrogen base growth medium (Sigma-Aldrich, St. Louis, MO, USA). Stock solutions (5000 μg/mL) of wintergreen essential oils were prepared in DMSO and diluted as above. The freshly harvested fungi with approximately 7.5107 CFU/mL final concentration were added to each well of 96-well microdilution plates and were incubated at 35 °C for 24 h. DMSO and amphotericin B (Sigma-Aldrich, St. Louis, MO, USA) were negative and positive antifungal control, respectively [15,20].

## 4. Conclusions

The *Gaultheria fragrantissima* essential oils from different geographical locations and varying distillation conditions showed almost similar results on chemical compositions and enantiomeric distributions of chiral terpenoids. The highest extraction yield was observed in dry leaves (W6), at 1.48%. The yield of extraction increased drastically by soaking in 5% sodium chloride solution. The major compound in wintergreen essential oil was methyl salicylate, an ester of salicylic acid which was above 99%. The optimum time to obtain the maximum number of compounds in wintergreen oil was 120 min of distillation time. Elsholtzia ketone and β-dehydroelsholtzia ketone were detected in Chinese but not in Nepalese wintergreen essential oil. This result may be used to distinguish the origin of the essential oil in either Nepal or China. Methyl salicylate synthesized using aspirin showed the presence of ethyl salicylate and the absence of dimethyl 2-hydroxyterephthalate, a synthetic marker compound. Thus, marker-based analysis using GC–MS alone is insufficient for adulteration detection, but in combination with chiral GC–MS and ^14^C analysis, it will be a powerful technique in adulteration detection. Altogether, 10 chiral compounds, linalool, α-terpineol, β-caryophyllene, α-pinene, β-pinene, limonene, germacrene D, β-phellandrene, camphene, and camphor, were detected for the first time. Our experiments show that geographical locations and varying distillation conditions do not affect the enantiomeric distribution of the chiral compounds. The range of enantiomeric distribution of chiral terpenoids may be used in adulteration detection. Besides these, Solukhumbu (W1), Champadevi (W3), and Sundarijal (W4) samples showed antibacterial activity against *Staphylococcus epidermidis* with an MIC value of 156.3 μg/mL. Similarly, W1 showed effectiveness against *Aspergillus niger* (MIC = 78.1 μg/mL).

## Figures and Tables

**Table 1:** Yields of natural wintergreen oil and synthetic methyl salicylate.

***Gaultheria fragrantissima* Leaves**	**% Yields**	**vs.**	**Synthetic Methyl Salicylate**	**% Yields**
Solukhumbu (W1)	0.27%		Aspirin tablet as precursor (W9)	52.5%
Syangja (W2)	0.57%		Salicylic acid as precursor (W10)	66.7%
Champadevi (W3)	0.70%			
Sundarijal (W4)	0.60%			
Fresh leaves (W5)	0.59%			
Dry leaves (W6)	1.48%			
18 h soaked (W7)	1.00%			
18 h soaked in 5% NaCl (W8)	1.23%			

**Table 2:** Compositional analysis of wintergreen essential oil from different geographical locations ^a^; “-” indicates not detected and “t” indicates trace (<0.01%).

RRI	Compounds	W1	W2	W3	W4
800	*n*-Octane	-	-	t	-
802	Hexanal	-	-	t	-
848	(3*Z*)-Hexenol	-	0.04	0.08	0.05
862	1-Hexanol	-	t	t	-
931	α-Pinene	-	0.01	-	-
1005	(3*Z*)-Hexenyl acetate	-	0.05	0.13	0.05
1007	Hexyl acetate	-	-	t	-
1027	Limonene	-	0.01	-	-
1069	1-Octanol	-	-	t	-
1098	Linalool	0.02	t	0.02	0.03
1103	Nonanal	-	t	0.01	-
1155	Methyl *p*-salicylate	-	0.01	-	-
1196	Methyl salicylate	99.89	99.78	99.69	99.84
1198	Methyl chavicol	-	-	-	0.01
1217	β-Cyclocitral	-	-	t	-
1248	Geraniol	-	-	t	-
1260	(2*E*)-Decenal	-	-	t	-
1266	Ethyl salicylate	0.06	0.01	-	-
1348	Eugenol	0.03	0.06	0.05	0.02
1379	Methyl *p*-mercaptobenzoate	-	0.01	t	-
1417	β-Caryophyllene	-	t	t	-
1480	Germacrene D	-	-	t	-
1574	Caryophyllene oxide	-	-	t	-
1663	(3*Z*)-Hexenyl salicylate	-	t	-	-
1864	Benzyl salicylate	-	t	-	-
2002	Manoyl oxide	-	-	0.02	-
2011	13-*epi*-Manool oxide	-	0.02	-	-
2049	Abietatriene	-	t	t	-
2061	Phenylethyl alcohol dimer	-	t	-	-
2300	*n*-Tricosane	-	-	t	-
2700	*n*-Heptacosane	-	-	t	-

^a^ Sample collections from Nepal: Solukhumbu (W1), Syangja (W2), Champadevi (W3), and Sundarijal (W4).

**Table 3:** Compositional analysis of wintergreen essential oil based on distillation time (in minutes); “-” indicates not detected and “t” indicates trace (<0.01%).

RRI	Compounds	30 min	60 min	120 min	180 min
759	Pentanol	-	t	0.01	-
783	Prenol	-	-	t	-
825	2,6-Dimethyl heptane	-	-	t	-
848	(3*Z*)-Hexenol	0.15	0.26	0.31	0.28
859	(2*E*)-Hexenol	-	t	t	-
862	1-Hexanol	0.01	0.02	0.03	0.02
960	Benzaldehyde	-	t	-	-
974	1-Octen-3-ol	t	-	-	-
990	6-Methyl-5-hepten-2-ol	t	t	t	-
1005	(3*Z*)-Hexenyl acetate	0.12	0.04	0.02	0.06
1007	Hexyl acetate	t	-	-	-
1017	3-Methyl-1,2-cyclopentanedione	t	t	t	-
1033	Benzyl alcohol	t	0.01	0.01	t
1048	*o*-Cresol	-	t	-	-
1069	1-Octanol	t	t	t	t
1098	Linalool	0.01	t	0.01	0.03
1103	Nonanal	t	t	t	0.01
1196	Methyl salicylate	99.66	99.61	99.52	99.26
1248	Geraniol	-	-	t	-
1260	(2*E*)-Decenal	-	t	-	-
1266	Ethyl salicylate	0.01	0.01	0.02	0.01
1348	Eugenol	0.02	0.03	0.06	0.10
1379	Methyl *p*-mercaptobenzoate	-	t	-	-
1384	β-Bourbonene	-	-	-	t
1417	β-Caryophyllene	t	-	-	t
1480	Germacrene D	t	-	-	t
1864	Benzyl salicylate	-	0.01	-	-
2002	Manoyl oxide	0.02	-	0.01	0.13
2011	13-*epi*-Manool oxide	-	0.01	-	-
2049	Abietatriene	-	-	t	0.1
2300	*n*-Tricosane	-	-	t	-
2500	*n*-Pentacosane	-	-	t	-

**Table 4:** Compositional analysis of fresh (W5), dry (W6), 18 h soaked (W7), and soaked in 5% sodium chloride solution (W8) wintergreen leaf essential oils; “MS” indicates mass detector integration (%), “FID” indicates flame ionization detector integration (%), “-” indicates not detected, and “t” indicates trace (<0.01%).

RRI	Compounds	W5MS	W5FID	W6MS	W6FID	W7MS	W7FID	W8MS	W8FID
848	(3*Z*)-Hexenol	0.34	0.39	0.04	0.05	0.11	0.17	0.37	0.56
859	(2*E*)-Hexenol	-	-	-	-	-	-	t	0.01
862	1-Hexanol	0.04	0.05	0.01	0.013	0.01	0.03	0.04	0.08
931	α-Pinene	t	0.01	-	-	-	-	-	-
948	Camphene	0.01	0.02	-	-	-	-	-	-
960	Benzaldehyde	-	-	0.01	0.01	-	-	-	-
990	6-Methyl-5-hepten-2-ol	-	-	t	t	-	-	-	-
1005	(3*Z*)-Hexenyl acetate	0.02	0.02	-	-	t	0.01	0.01	0.01
1017	3-Methyl-1,2-cyclopentanedione	t	0.01	t	0.01	0.02	0.06	-	-
1027	Limonene	0.02	0.02	0.02	0.02	0.01	0.02	t	0.02
1029	β-Phellandrene	0.01	0.01	-	-	-	-	-	-
1031	1,8-Cineole	t	t	-	-	-	-	-	-
1033	Benzyl alcohol	-	-	0.01	0.02	-	-	-	-
1067	*cis*-Linalool oxide (furanoid)	-	-	-	-	t	0.01	-	-
1069	1-Octanol	0.01	0.01	0.01	0.01	-	-	-	-
1098	Linalool	0.01	0.01	0.02	0.02	0.01	0.01	0.01	0.01
1103	Nonanal	0.01	0.01	0.01	0.01	t	t	t	0.01
1169	Ethyl benzoate	0.01	0.01	-	-	0.02	0.02	t	0.01
1196	Methyl salicylate	99.33	99.28	99.51	99.56	99.57	99.40	99.53	99.21
1217	β-Cyclocitral	-	-	-	-	t	0.01	t	-
1229	(3*Z*)-Hexenyl 2-methylbutanoate	t	t	-	-	-	-	-	-
1248	Geraniol	-	-	0.01	0.01	-	-	-	-
1260	(2*E*)-Decenal	t	t	t	t	-	-	-	-
1266	Ethyl salicylate	0.11	0.08	0.02	0.01	0.21	0.20	0.02	0.03
1268	(*E*)-Cinnamaldehyde	-	-	0.01	0.01	-	-	-	-
1315	Methyl *o*-mercaptobenzoate	-	-	0.01	0.01	-	-	-	-
1348	Eugenol	0.06	0.06	0.19	0.15	0.02	0.04	0.02	0.05
1379	Methyl *p*-mercaptobenzoate	t	t	0.01	0.01	-	-	-	-
1417	β-Caryophyllene	-	-	t	t	t	t	-	-
1436	Aromadendrene	-	-	-	-	t	t	-	-
1445	Geranyl acetone	-	-	0.01	t	-	-	-	-
1476	(*E*)-β-Ionone	-	-	t	0.01	-	-	-	-
1480	Germacrene D	-	-	-	-	0.01	0.01	-	-
1563	(3*Z*)-Hexenyl benzoate	t	0.01	t	0.01	-	-	-	-
1663	(3*Z*)-Hexenyl salicylate	0.01	0.01	0.03	0.03	-	-	-	-
1674	Hexyl salicylate	-	-	0.01	0.01	-	-	-	-
1864	Benzyl salicylate	0.01	0.01	0.04	0.03	-	-	-	-
2002	Manoyl oxide	-	-	-	-	0.01	0.01	t	0.01
2011	13-*epi*-Manool oxide	-	-	0.02	0.01	-	-	-	-

**Table 5:** Comparison of Nepalese and Chinese commercial wintergreen oils; “nd” indicates not detected and “t” indicates trace (<0.01%).

RRI	Compounds	Nepalese Commercial Wintergreen Oil	Chinese Commercial Wintergreen Oil
800	*n*-Octane	nd-t	nd
802	Hexanal	nd-t	nd
839	5-*t*-Butylcyclopentadiene	nd-t	nd
848	(3*Z*)-Hexenol	nd-t	nd-0.09
859	(2*E*)-Hexenol	nd	nd-t
862	1-Hexanol	nd-t	nd-0.05
891	Styrene	nd-t	nd
931	α-Pinene	nd-0.03	nd-0.06
948	Camphene	nd-0.01	nd-0.01
960	Benzaldehyde	nd-0.01	nd
971	Sabinene	nd	nd-0.01
972	Phenol	nd-0.03	nd-0.01
976	β-Pinene	nd-0.02	nd-0.02
988	Myrcene	nd	nd-0.01
995	δ-2-Carene	nd-t	nd
1002	α-Phellandrene	nd-0.01	nd
1004	(3*Z*)-Hexenyl acetate	nd	nd-0.01
1014	α-Terpinene	nd	nd-t
1017	3-Methyl-1,2-cyclopentanedione	nd-t	nd-0.02
1023	*p*-Cymene	t-0.01	nd-t
1027	Limonene	t-0.01	nd-0.02
1031	1,8-Cineole	nd-0.01	t-0.06
1033	Benzyl alcohol	nd-0.01	nd-0.01
1055	γ-Terpinene	nd-t	nd-t
1067	*cis*-Linalool oxide (furanoid)	nd	nd-t
1069	1-Octanol	nd-t	nd-t
1081	*trans*-Linalool oxide (furanoid)	nd	nd-t
1085	Terpinolene	nd-t	nd-t
1091	*p*-Cymenene	nd-t	nd
1098	Linalool	0.02–0.03	0.01–0.10
1102	Hotrienol	nd-t	nd
1103	Nonanal	nd-0.01	nd-0.01
1143	Camphor	nd	nd-0.01
1169	Ethyl benzoate	nd-t	nd
1176	Terpinen-4-ol	nd	nd-0.01
1196	Methyl salicylate	99.54–99.86	99.42–99.91
1199	Elsholtzia ketone	nd	nd-0.01
1239	Neral	nd	nd-t
1244	Carvone	nd-0.01	nd-0.01
1248	Geraniol	nd-t	nd
1266	Ethyl salicylate	0.05–0.32	0.01–0.09
1281	Vitispirane	nd-0.01	0.01–0.03
1282	Bornyl acetate	nd-t	nd
1293	Methyl naphthalene	nd	nd-t
1299	β-Dehydroelsholtzia ketone	nd	nd-0.01
1300	Carvacrol	nd-0.01	nd
1348	Eugenol	0.04–0.12	nd-0.07
1367	α-Ylangene	nd	nd-0.01
1373	α-Copaene	nd	nd-t
1379	Methyl *p*-mercaptobenzoate	nd-0.01	nd-0.01
1417	β-Caryophyllene	nd-0.01	nd-0.01
1452	α-Humulene	nd	nd-t
1568	(3*Z*)-Hexenyl benzoate	nd	nd-0.01
2011	13-*epi*-Manool oxide	nd-0.01	nd
2061	Phenylethyl alcohol dimer	nd	nd-t

**Table 6:** Comparison of volatile compounds present in commercial methyl salicylate with methyl salicylate synthesized from aspirin and synthesized from salicylic acid ^a^; “-” indicates not detected and “t” indicates trace (<0.01%).

RRI	Compounds	W9	W10	W11
846	Dimethyl sulfate	0.02	0.02	-
972	Phenol	-	-	0.02
1196	Methyl salicylate	98.82	99.64	99.65
1266	Ethyl salicylate	0.31	-	-
1301	Isopropyl salicylate	0.01	-	-
1330	Methyl *o*-anisate	t	0.01	0.01
1448	Methyl paraben	-	-	0.01
1514	Ethyl methyl phthalate	0.34	-	-
1604	Dimethyl 2-hydroxyterephthalate	-	t	0.29
1897	Diisobutyl phthalate	0.01	0.01	0.01
2061	Phenylethyl alcohol dimer	0.42	0.3	t
	Total Identified	99.93%	99.98%	100%

^a^ W9 = methyl salicylate synthesized from aspirin; W10 = methyl salicylate synthesized from salicylic acid; W11 = commercial methyl salicylate.

**Table 7:** Comparison of natural and adulterate wintergreen essential oil; “-” indicates not detected and “t” indicates trace (<0.01%).

RRI	Compounds	Natural Wintergreen Oil ^a^	Addition of Commercial Methyl Salicylate in Natural Wintergreen Oil (%)			
			20	30	40	50
800	*n*-Octane	t	t	t	t	t
802	Hexanal	t	t	t	t	t
848	(3*Z*)-Hexenol	0.12	0.09	0.08	0.06	0.05
862	1-Hexanol	0.07	0.05	0.04	0.03	0.03
900	*n*-Nonane	t	t	t	t	t
931	α-Pinene	0.03	0.02	0.02	0.01	0.01
948	Camphene	t	t	t	t	t
960	Benzaldehyde	t	t	t	t	-
972	Phenol	-	0.01	0.01	0.01	0.02
976	β-Pinene	0.01	t	t	t	t
988	2-Pentylfuran	-	-	t	-	-
1005	(3*Z*)-Hexenyl acetate	-	t	t	t	-
1017	3-Methyl-1,2-cyclopentanedione	0.01	0.01	0.01	0.01	0.01
1023	*p*-Cymene	0.01	0.01	t	t	t
1027	Limonene	0.01	t	t	t	t
1029	β-Phellandrene	-	-	-	-	t
1031	1,8-Cineole	0.03	0.02	0.02	0.01	0.01
1033	Benzyl alcohol	-	0.01	0.01	t	t
1067	*cis*-Linalool oxide (furanoid)	t	t	-	-	-
1069	1-Octanol	-	-	t	-	-
1070	*cis*-Sabinene hydrate	-	-	-	t	-
1098	Linalool	0.05	0.04	0.03	0.03	0.02
1103	Nonanal	t	t	t	t	t
1196	Methyl salicylate	99.46	99.5	99.5	99.53	99.5
1217	β-Cyclocitral	t	t	t	t	t
1223	Nerol	-	t	-	-	-
1229	(3*Z*)-Hexenyl 2-methylbutanoate	-	t	-	t	-
1248	Geraniol	0.03	0.03	0.03	0.03	0.02
1260	(2*E*)-Decenal	-	t	t	t	-
1266	Ethyl salicylate	0.05	0.05	0.04	0.04	0.03
1281	Vitispirane	0.02	0.01	0.01	0.01	0.01
1299	β-Dehydroelsholtzia ketone	0.04	0.03	0.03	0.02	0.02
1315	Methyl *o*-mercaptobenzoate	-	t	t	t	t
1330	Methyl *o*-anisate	-	t	t	0.01	0.01
1348	Eugenol	0.02	0.02	0.02	0.01	0.01
1379	Methyl *p*-mercaptobenzoate	0.01	0.01	0.01	0.01	0.01
1384	β-Bourbonene	t	t	-	-	-
1417	β-Caryophyllene	0.02	0.01	0.01	0.01	0.01
1431	*trans*-α-Bergamotene	t	t	-	-	t
1436	Aromadendrene	t	t	t	-	-
1448	Methyl paraben	-	t	t	t	0.01
1476	(*E*)-β-Ionone	-	t	t	t	-
1480	Germacrene D	t	t	t	-	-
1502	(*E*,*E*)-α-Farnesene	-	t	-	-	-
1505	β-Bisabolene	t	t	-	t	-
1516	δ-Cadinene	-	t	-	-	-
1568	(3*Z*)-Hexenyl benzoate	0.01	0.01	0.01	0.01	0.01
1575	Hexyl benzoate	t	t	-	-	-
1604	Dimethyl 2-hydroxyterephthalate	-	0.07	0.12	0.16	0.21
1663	(3*Z*)-Hexenyl salicylate	t	t	t	t	t
1674	Hexyl salicylate	-	t	-	-	-
1864	Benzyl salicylate	-	t	t	-	-
2061	Phenylethyl alcohol dimer	t	-	t	t	t

^a^ Source: Aromatic Plant Research Center (Lehi, UT, USA).

**Table 8:** Enantiomeric distributions of chiral terpenoids present in wintergreen essential oil based on geographical location; “–” indicates not detected; “+” indicates dextrorotatory.

Chiral Compounds	Enantiomeric Distribution, Dextrorotatory (+) and Levorotatory (−)							
	(W1)		(W2)		(W3)		(W4)	
	(+)	(−)	(+)	(−)	(+)	(−)	(+)	(−)
Linalool	56.2	43.8	58.3	41.7	50.3	49.7	53.4	46.6
α-Terpineol	–		–		23.8	76.2	31.0	69.0
β-Caryophyllene	–		–		0.0	100.0	–	
α-Pinene	–		100.0	0.0	–		–	
Limonene	100.0	0.0	100.0	0.0	–		–	
Camphor	100.0	0.0	–		–		100.0	0.0

**Table 9:** Enantiomeric distributions of chiral terpenoids present in wintergreen essential oil based on distillation time; “–” indicates not detected; “+” indicates dextrorotatory.

Chiral Compounds	Enantiomeric Distribution, Dextrorotatory (+), and Levorotatory (−)							
	30 min		60 min		120 min		180 min	
	(+)	(−)	(+)	(−)	(+)	(−)	(+)	(−)
Linalool	62.3	37.7	52.9	47.1	55.1	44.9	51.9	48.1
α-Terpineol	26.1	73.9	29.4	70.6	26.1	73.9	34.1	65.9
β-Caryophyllene	0.0	100.0	0.0	100.0	–		0.0	100.0
Camphor	–		100.0	0.0	–		–	

**Table 10:** Enantiomeric distributions of chiral terpenoids present in wintergreen essential oil based on different distillation conditions; “–” indicates not detected; “+” indicates dextrorotatory.

Chiral Compounds	Enantiomeric Distribution, Dextrorotatory (+), and Levorotatory (−)							
	W5		W6		W7		W8	
	(+)	(−)	(+)	(−)	(+)	(−)	(+)	(−)
Linalool	55.1	44.9	61.7	38.3	56.9	43.1	57.6	42.4
α-Terpineol	33.1	66.9	31.9	68.1	32.6	67.4	31.6	68.4
Limonene	100.0	0.0	100.0	0.0	100.0	0.0	100.0	0.0
Camphene	100.0	0.0	–		–		–	
β-Phellandrene	100.0	0.0	–		–		–	
Germacrene D	–		–		100.0	0.0	–	

**Table 11:** Enantiomeric distributions of chiral terpenoids present in commercial wintergreen from Nepal and China; “–” indicates not detected; “+” indicates dextrorotatory.

Chiral Compounds	Enantiomeric Distribution, Dextrorotatory (+), and Levorotatory (−)			
	Nepal		China	
	(+)	(−)	(+)	(−)
Linalool	50.6–59.4	40.6–49.4	47.3–54.7	45.3–52.7
α-Terpineol	31.8–32.7	67.3–68.2	32.9–35.2	64.8–67.1
Camphor	100.0	0.0	–	
β-Caryophyllene	0.0	100.0	–	
β-Pinene	0.0	100.0	–	
α-Pinene	56.6–61.6	38.4–43.4	59.4–60.9	39.1–40.6

**Table 12:** Minimum inhibitory concentrations (MICs) of wintergreen essential oil against tested bacterial strains ^a^.

Name of Bacteria	MICs (μg/mL)		
	(W1)	(W3)	(W4)
*Bacillus cereus*	625	625	625
*Propionibacterium acnes*	312.5	312.5	312.5
*Pseudomonas aeruginosa*	625	312.5	625
*Serratia marcescens*	625	625	625
*Staphylococcus aureus*	1250	1250	312.5
*Staphylococcus epidermidis*	156.3	156.3	156.3
*Streptococcus pyogenes*	625	625	625

^a^ Essential oil samples from Solukhumbu (W1), Champadevi (W3), and Sundarijal (W4).

**Table 13:** Minimum inhibitory concentrations (MICs) of wintergreen essential oil against tested fungal strains ^a^.

Fungal Strains	MICs (μg/mL)		
	W1	W3	W4
*Aspergillus niger*	78.1	156.3	156.3
*Aspergillus fumigatus*	312.5	312.5	312.5
*Candida albicans*	625	312.5	312.5
*Microsporum canis*	156.3	156.3	312.5
*Microsporum gypseum*	312.5	312.5	312.5
*Trichophyton mentagrophytes*	312.5	312.5	156.3
*Trichophyton rubrum*	312.5	156.3	312.5

^a^ Essential oil samples from Solukhumbu (W1), Champadevi (W3), and Sundarijal (W4).

## Data Availability

Not applicable.
